# Effect of ADH II Deficiency on the Intracellular Redox Homeostasis in *Zymomonas mobilis*

**DOI:** 10.1100/2012/742610

**Published:** 2012-05-03

**Authors:** Nina Galinina, Zane Lasa, Inese Strazdina, Reinis Rutkis, Uldis Kalnenieks

**Affiliations:** Institute of Microbiology and Biotechnology, University of Latvia, Kronvalda Boulevard 4, 1586 Riga, Latvia

## Abstract

Mutant strain of the facultatively anaerobic, ethanol-producing bacterium *Zymomonas mobilis*, deficient in the Fe-containing alcohol dehydrogenase isoenzyme (ADH II), showed impaired homeostasis of the intracellular NAD(P)H during transition from anaerobic to aerobic conditions, and also in steady-state continuous cultures at various oxygen supplies. At the same time, ADH II deficiency in aerobically grown cells was accompanied by a threefold increase of catalase activity and by about 50% increase of hydrogen peroxide excretion. It is concluded that ADH II under aerobic conditions functions to maintain intracellular redox homeostasis and to protect the cells from endogenous hydrogen peroxide.

## 1. Introduction

*Zymomonas mobilis* is a facultatively anaerobic, obligately fermentative Gram-negative bacterium with a very potent homoethanol fermentation pathway. Ethanol productivity of this bacterium, exceeding by 3–5-fold that of yeast [1], in combination with high ethanol yield and ethanol tolerance makes *Z. mobilis* one of the most promising producer microorganisms for bioethanol [2, 3]. Most of metabolic engineering work with this bacterium is focussed upon broadening of its substrate spectrum beyond glucose, fructose, and sucrose, with an emphasis on the ability to produce ethanol from lignocellulosic substrates [4–7]. The fermentation pathway in *Z. mobilis* is terminated by two NAD^+^-dependent alcohol dehydrogenase (ADH) isoenzymes—ADH I (*adhA*) and ADH II (*adhB*) [8, 9]. Although the ADH isoenzymes have been thoroughly characterised [10, 11] and the iron-containing ADH II has been widely used in metabolic engineering of ethanol producer strains [2], the specific functions of each isoenzyme in growing *Z. mobilis* have not been fully revealed.

 Apart from its ethanol fermentation pathway, *Z. mobilis* possesses an active aerobic respiratory chain [12, 13], with oxygen uptake rates substantially higher than those of *S. cerevisiae* or *E. coli* [14, 15]. There is only one functional respiratory NAD(P)H dehydrogenase in *Z. mobilis* electron transport chain, belonging to the type II (*ndh*) respiratory dehydrogenase family [16–18]. A *bd*-type quinol oxidase is the only terminal oxidase of *Z. mobilis* identified so far [15, 18, 19]. The Krebs cycle in *Z. mobilis* is truncated and does not function as a catabolic pathway [18–20], therefore respiratory chain competes with ethanol synthesis for the reducing equivalents, coming from the Entner-Doudoroff (ED) glycolysis [21, 22], which is thus the sole source of NAD(P)H in the central catabolism.

**Figure 1:**
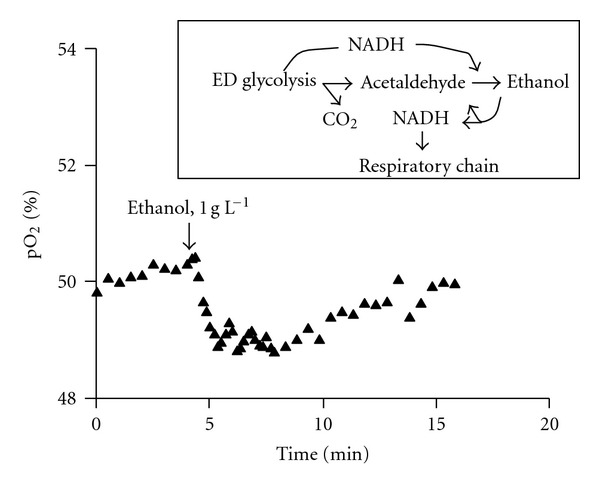
Typical response of aerobic chemostat culture of *Zymomonas mobilis* Zm6 to ethanol addition. Cultivation parameters: 1 L culture volume, pH 5.5, D 0.2 h^−1^, air flow 2 L min^−1^, stirring rate 500–600 r.p.m. A burst of respiration, seen as a transient decrease of pO_2_ in the fermentor, takes place after addition of 1 g L^−1^ ethanol. *Inset*. The proposed scheme of generation and utilization of reducing equivalents in the ethanol cycle—two opposite, rapid ADH reactions, resulting in a slow net aerobic ethanol synthesis.

 Competition between respiration and ethanol synthesis leads to a specific steady state of aerobic continuous culture, in which one of the ADH isoenzymes catalyzes oxidation of ethanol, while the other one catalyzes ethanol synthesis (see Figure 1, inset), together forming an “ethanol cycle” [23, 24]. Perturbation of this steady state indicated that the two opposite ADH reactions proceed several times faster than the net synthesis of ethanol. Addition of a small pulse of ethanol to chemostat culture caused a short, rapid burst of ethanol oxidation (Figure 1) against the background of slow net aerobic ethanol synthesis. Notably, the ADH II-deficient mutant under aerobic steady state had lost the ability to oxidize the added ethanol [24], although the activity of the other ADH isoenzyme was high. The model assumes that each isoenzyme operates in a different redox microenvironment, for simultaneous catalysis of opposite redox reactions to be thermodynamically feasible.

In the present work we studied some properties of aerobic metabolism of the ADH II-negative *Z. mobilis* strain, to gain more insight in the putative function of ADH II in the “ethanol cycle.” The results demonstrate (i) a role of ADH II in the regulation of intracellular NAD(P)H pools in *Z. mobilis*, both over time intervals of several minutes (comparable to the relaxation time of perturbed “ethanol cycle,” see Figure 1), as well as under steady-state conditions during continuous cultivation, and (ii) a relation of ADH II to the mechanisms of removal of endogenous hydrogen peroxide.

## 2. Materials and Methods

### 2.1. Bacterial Strains, Cultivation, and Preparation of Membranes

Two strains of *Zymomonas mobilis* were used: ATCC 29191 (Zm6) and ADH II-deficient mutant (adhB-), constructed previously [24] by insertion of kanamycin-resistance determinant into the *adhB* locus of strain Zm6 by means of homologous recombination. In the mutant strain the activity of ADH II was close to zero, while the activity of ADH I remained similar to that in the parent strain [24].

 Both strains were maintained and cultivated on liquid medium containing glucose (50 g L^−1^), yeast extract (5 g L^−1^), potassium dihydrogen phosphate (1 g L^−1^), ammonium sulfate (1 g L^−1^), and magnesium sulfate (0.5 g L^−1^), pH 5.5. For preparation of inoculum and during the culture maintenance, kanamycin (250 mg L^−1^) was added to the growth medium of the mutant strain. Cells, needed for experiments with nongrowing cell suspensions and for preparation of membranes, were cultivated in the batch mode overnight under oxygen-limited conditions (0.4 to 0.5 L of culture in 0.5 L flasks without shaking) at 30°C and were referred to as “anaerobically grown cells.” Aerobic batch cultivation was done overnight in 750 mL shaken flasks, containing 100 mL of culture, on a shaker at 200 r.p.m. and 30°C. Continuous cultivation was carried out in a Labfors fermenter (Infors), of 1 L working volume, at 30°C, pH 5.5, and 0.25 h^−1^ dilution rate. The growth medium contained 20 g L^−1^ glucose, 5 g L^−1^ yeast extract and mineral salts, as described above. Growth was initiated under anaerobic conditions, gassing the culture with nitrogen at 0.4 L min^−1^ flow rate. After anaerobic steady state was reached, aeration of the culture was started and gradually increased, until aerobic steady state was established, with the air flow around 2 L min^−1^ and stirring rate 550 r.p.m.

 For preparation of cytoplasmic membrane vesicles, cells were sedimented by centrifugation at 5000 r.p.m. for 15 min and resuspended in 100 mM potassium phosphate buffer, containing 2 mM magnesium sulfate, pH 6.9. Disruption of cells by ultrasonic disintegration and separation of cytoplasmic membranes by ultracentrifugation was performed as described previously [12]. The final concentration of membrane preparations was in the range of 4.0 to 5.0 mg protein mL^−1^.

### 2.2. Luminometric Determination of NAD(P)H

NAD(P)H concentrations were determined with an LKB “Wallac 1251” luminometer, using the “Roche” bacterial luciferase assay, basically following the standard protocol [25]. 1 mL sample of cell suspension was quenched with 30 *μ*L 6 M potassium hydroxide. Before determination of the total reduced dinucleotide content, the quenched sample was diluted hundredfold with 100 mM potassium phosphate buffer, pH 7.0. For determination of NADPH, the quenched sample was first diluted tenfold with the buffer, then 10 *μ*L of 10 mM pyruvate and 5 *μ*L of lactate dehydrogenase were added to a 100 *μ*L aliquot of the diluted sample in order to oxidize NADH, and finally, the mixture was diluted tenfold repeatedly with the same buffer. Concentrations of the reduced nicotinamide dinucleotides were calculated by reference to a calibration curve. NADH concentration of the sample was found by subtraction of the estimated NADPH concentration from the total reduced dinucleotide concentration.

### 2.3. Analytical Methods

“Radiometer” Clark-type oxygen electrode was used for oxygen consumption rate (Q_O_2__) assays for whole cells and membrane preparations. Oxygen consumption by membranes was measured in a reaction mixture, containing 100 *μ*L of membrane preparation and 400 *μ*L of 50 mM potassium phosphate buffer, pH 6.9, with added NADH or NADPH at 1 mM final concentration. Carbon dioxide production by cell suspensions was monitored with “Radiometer” CO_2_ electrode. Hydrogen peroxide generation was determined by quenching of scopoletin fluorescence in the presence of horseradish peroxidase [26], using fluorimeter FluoroMax-3 “Jobin Yvon.” The reaction of hydrogen peroxide generation was initiated by addition of 2.5 mM (final concentration) glucose to cell suspension in 100 mM potassium phosphate buffer with 2 mM EDTA, pH 7.6, and fluorescence was monitored at 350 nm excitation and 460 nm emission wavelengths. Fluorescence quenching was calibrated by addition of small known amounts of hydrogen peroxide to the reaction mixture. Catalase activity was measured in cell-free extracts in the same buffer. Reaction was initiated by addition of 20 mM H_2_O_2_ (final concentration), and decomposition of hydrogen peroxide was monitored as absorbance decreases at 240 nm [27]. Protein concentration in membrane samples was determined according to Markwell et al. [28]. Cell concentration was determined as OD_550_, and dry cell mass of the suspensions was calculated using a calibration curve. All results are mean values of at least three independent experiments. Standard errors are given in brackets or as error bars.

## 3. Results

### 3.1. Effect of ADH II Deficiency on the Intracellular NAD(P)H Levels

**Figure 2:**
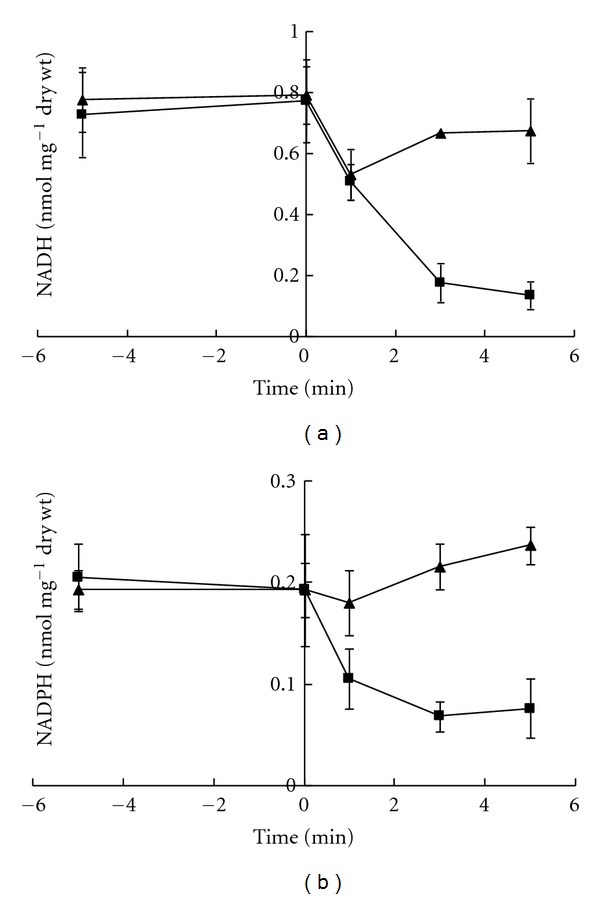
Time course of the intracellular NADH (a) and NADPH (b) pools in glycolysing cell suspensions after switching from anaerobic incubation to aeration (at zero time); (▲) Zm6, (■) adhB-. Cells were grown overnight without aeration, washed, resuspended at 7 mg dry wt mL^−1^ in 50 mM potassium phosphate buffer, pH 6.9, and gassed with oxygen-free nitrogen (2 L min^−1^ flow through 50 mL of cell suspension). Glucose was added 10 minutes before the start of aeration, at 10 g L^−1^ final concentration. Aerobic conditions were set by switching from oxygen-free nitrogen to gassing with air at 2 L min^−1^ flow rate.

Short-term response of *Zymomonas mobilis* intracellular NADH and NADPH concentrations was monitored upon transition of cells from anaerobic to aerobic conditions. Cells grown overnight without aeration (in a thermostat without shaking) were washed and resuspended at 7 mg dry wt mL^−1^ in 50 mM potassium phosphate buffer, pH 6.9. Suspensions were gassed with oxygen-free nitrogen (2 L min^−1^) for 5 minutes, then glucose was added to the final concentration of 10 g L^−1^, and gassing was continued for 10 minutes. Samples for NAD(P)H assays were taken at 5-minute intervals. Immediately after the second sampling (which was done at the 10th minute after glucose addition, corresponding to zero time in Figure 2) nitrogen gassing was replaced by aeration, and samples were taken after 1, 3, and 5 minutes.

**Figure 3:**
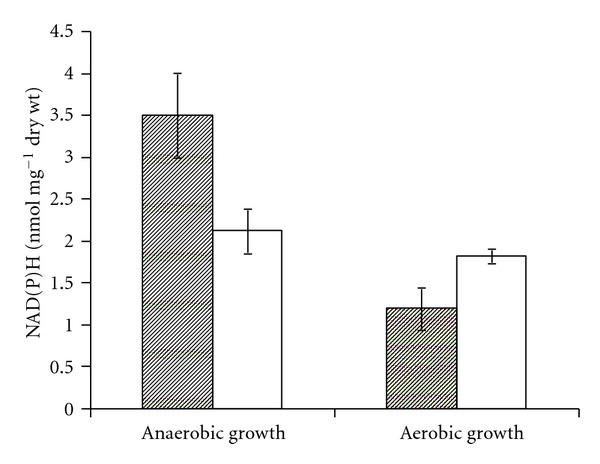
Intracellular NAD(P)H concentration in aerobic and anaerobic steady-state chemostat cultures; empty bars—Zm6, filled bars—adhB-. The total of both reduced cofactor concentrations is given. Continuous cultivation parameters were as described in Section 2.

The mean values of four independent experiments are shown in Figure 2. During the 5 minutes of anaerobic glucose consumption both strains built up closely similar intracellular NADH (Figure 2(a)) and NADPH (Figure 2(b)) pools. Yet, switching to aeration revealed large differences between the wild-type and the ADH II-deficient phenotype. In Zm6, aeration caused a transient perturbation of the reduced cofactor levels. The initial slight decrease of NAD(P)H during the first minute was followed by a rapid return to the near-anaerobic concentration range. A much more pronounced decrease of NAD(P)H pools took place in the ADH II-deficient strain. It appeared that by eliminating of the ADH II activity the cells were turned unable to keep the reduced nicotinamide nucleotide pools stable during the onset of respiration. Furthermore, during steady-state continuous cultivation (Figure 3) the mutant strain under anaerobic conditions showed substantially higher NAD(P)H pools, than under aerobic conditions (*P* < 0.05). For the parent strain under similar culture conditions the reduced cofactor pools were maintained at intermediary levels, and the difference between anaerobic and aerobic NAD(P)H pools was not statistically significant.

Apart from the ADH reaction, there are two other major catabolic fluxes potentially affecting the steady state of NAD(P)H concentrations in *Zymomonas mobilis*. These are (i) generation of the reduced cofactors by the ED glycolysis and (ii) oxidation of NAD(P)H in the respiratory chain. We compared these two fluxes in Zm6 to those in adhB- during the onset of aeration in anaerobic cell suspensions. Oxygen consumption was measured immediately after a tenfold dilution of anaerobic, glucose-consuming cell suspensions with air-saturated phosphate buffer, containing 2 g L^−1^ glucose and 2 g L^−1^ ethanol. Both strains showed closely similar oxygen consumption rates (Table 1). The same was true for the specific rates of NADH oxidation in membrane preparations: no statistically significant differences were found between both strains. *Zymomonas mobilis* is one of the few known microorganisms in which the type II respiratory NADH dehydrogenase shows a fairly high NADPH-oxidizing activity in the physiological range of pH [16, 17, 29]. As seen from Table 1, in the membrane preparations obtained from both *Z. mobilis* strains the NADPH oxidase activity was similar. At pH 6.9, in both strains it reached almost half of the NADH oxidase activity. Likewise, the rates of aerobic glycolysis did not differ significantly (not shown). The fluxes in the Entner-Doudoroff pathway were compared between the two strains by measuring the relative rates of carbon dioxide production in cell suspensions, treated in the same way as described above for the oxygen consumption assay. The rate of carbon dioxide production in the ADH II-deficient strain was found to be 101.5 (±5.9) % of that in Zm6. Thus, apart from the ADH reaction, the other catabolic fluxes, producing or consuming NAD(P)H, were not altered in the mutant strain.

### 3.2. Catalase Activity and H_2_O_2_ Generation in the Mutant Strain

**Figure 4:**
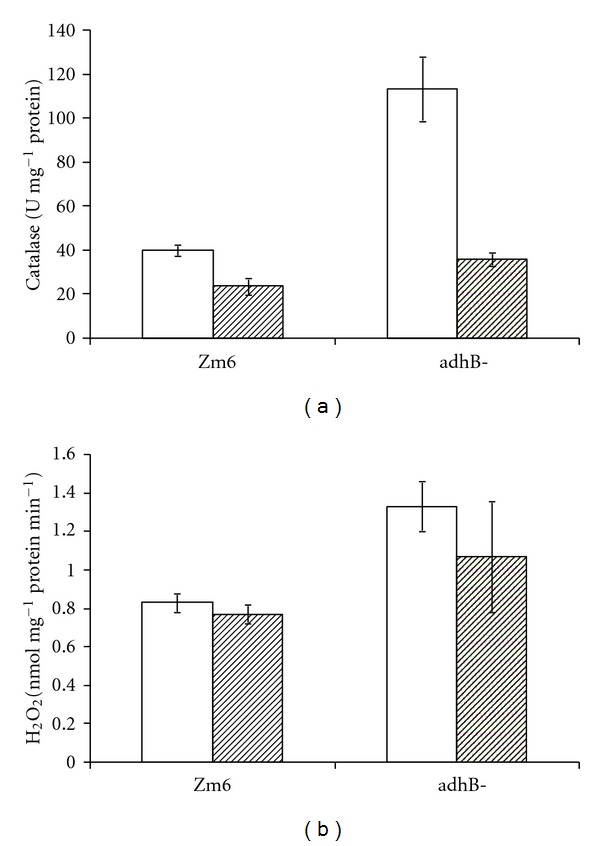
Catalase activity in cell-free extracts (a) and hydrogen peroxide production in stationary phase cell suspensions, supplied with 2.5 mM glucose (b); empty bars—aerobically grown cells, filled bars—anaerobically grown cells. Cell suspensions were obtained from anaerobic and aerobic overnight batch cultures. Cell-free extracts were prepared by ultrasonic disintegration.

It is well established that ADH II is a major stress protein in *Z. mobilis*, induced by high ethanol concentrations and elevated temperature [30]. Although ADH II itself is sensitive to oxygen due to presence of Fe^2+^ in its active site [31], participation of bacterial iron-containing ADH isoenzymes in the oxidative stress protection has been suggested previously. For *E. coli*, Echave et al. [32] showed deleterious effects of the iron-containing isoenzyme (AdhE) deficiency on aerobic growth, leading to morphologic defects and inability to grow aerobically on minimal media. They proposed that AdhE acts as an intracellular H_2_O_2_ scavenger, in particular at low or medium hydrogen peroxide concentrations. In order to find out if *Z. mobilis* ADH II also functions in the intracellular H_2_O_2_ turnover, we examined the excretion of hydrogen peroxide in the incubation medium of cell suspensions, as well as measured catalase activity in both strains (Figure 4). Although the transcription of catalase gene in *Z. mobilis* has been reported not to depend on aeration [33], catalase activity in aerobically cultivated parent strain was significantly higher (*P* < 0.05), than in anaerobically grown cells. Remarkably, under anaerobic batch culture conditions there was no statistically significant difference between the catalase activity in the parent and mutant strain, yet during aerobic batch cultivation catalase activity in the mutant strain dramatically increased, in the stationary phase cells exceeding that of the parent strain by a factor of three (Figure 4(a)). At the same time, aerobically grown glucose-consuming mutant cells excreted more hydrogen peroxide, in spite of the elevated catalase activity. The rate of hydrogen peroxide excretion in aerobically grown mutant cells was significantly higher than in Zm6 (*P* < 0.05). It exceeded the H_2_O_2_ excretion in the parent strain by about 50%, while no statistically significant difference in H_2_O_2_ production was found between anaerobically grown cells of both strains (Figure 4(b)).

## 4. Discussion

The presented data demonstrate that in *Z. mobilis* the iron-containing alcohol dehydrogenase isoenzyme is directly involved in the buffering of the reduced nicotinamide cofactor pools. We hypothesize that this physiological effect results from the operation of the “ethanol cycle” in respiring Zm6 cells, with ADH II stabilizing the NADH level at the expense of ethanol (re)oxidation. As both alcohol dehydrogenases are strictly NAD^+^ dependent [10, 11], most probably, the NADPH pool follows the shifts of the NADH pool due to some rapid redox-equilibrating reactions, like transhydrogenase. Such a redox-compensatory mechanism might be particularly efficient for the purpose of rapid, short-term regulation, before the tuning of metabolic fluxes at the transcriptional level takes place. However, ADH II deficiency affected the NAD(P)H homeostasis also in a longer timescale. During chemostat cultivations the mutant strain showed significantly altered steady-state levels of NAD(P)H, both under aerobic and anaerobic conditions, indicating a limited capacity of *Z. mobilis* to compensate for the ADH II deficiency.

 The ADH II deficiency did not hamper significantly the aerobic growth of *Z. mobilis* [24], in contrast to what has been reported for *E. coli* strain, deficient in the iron-containing alcohol dehydrogenase isoenzyme [32]. Yet, the mutant phenotype showed symptoms of altered turnover of the endogenous H_2_O_2_—a threefold increase of catalase activity and an elevated hydrogen peroxide excretion from aerobically cultivated cells. Apparently, AdhE of *E. coli* is not the only bacterial Fe-containing alcohol dehydrogenase with a peroxide stress-protective function. Little is known about the underlying mechanisms of this type of ADH function. In the light of our present findings it is tempting to think that ADH II might contribute to the peroxide stress protection via stabilization of the intracellular reduced nicotinamide cofactor pools, especially that of NADPH. High NADPH concentration is shown to be crucial in microorganisms under conditions of oxidative stress [34, 35], because NADPH supplies electrons to the cellular systems detoxifying the reactive oxygen species, including H_2_O_2_. Hence, stabilization of the NADPH pool by ADH II seems to be in line with the general oxidative stress-protection strategy of bacteria. Further studies will be necessary to verify the relation between the peroxide stress-protective and redox-stabilizing functions of ADH II.

## Figures and Tables

**Table 1:** Oxygen uptake rates of cells and cytoplasmic membrane vesicles. Cells grown overnight without aeration were used for the whole-cell experiments and membrane preparation. Oxygen uptake measurements in membrane preparations were carried out in 50 mM phosphate buffer, containing membranes at concentration of 0.4-0.5 mg protein mL^−1^, and 1.5 mM NADH or NADPH. For the whole-cell oxygen uptake measurements, 10 g L^−1^ glucose was added to anaerobic cell suspensions (7 mg dry wt mL^−1^ in 50 mM potassium phosphate buffer, pH 6.9). After 5 minutes of incubation the suspensions were diluted tenfold with air-saturated phosphate buffer, containing 2 g L^−1^ glucose and 2 g L^−1^ ethanol, and oxygen consumption rate was measured.

Strain	whole cells	membrane vesicles	
	Q_O_2__	Q_O_2__^NADH^	Q_O_2__^NADPH^/Q_O_2__^NADH^
	[U mg dry wt^−1^]	[U mg prot^−1^]	
Zm6	0.14 ± 0.02	0.29 ± 0.06	0.44 ± 0.13
adhB-	0.15 ± 0.02	0.22 ± 0.05	0.47 ± 0.12
